# Thrombin Induces Secretion of Multiple Cytokines and Expression of Protease-Activated Receptors in Mouse Mast Cell Line

**DOI:** 10.1155/2019/4952131

**Published:** 2019-11-14

**Authors:** Xiaobin Fang, Ren Liao, Yingyan Yu, Jingyi Li, Zaipei Guo, Tao Zhu

**Affiliations:** ^1^Department of Anesthesiology, West China Hospital of Sichuan University, Chengdu 610041, China; ^2^Department of Dermatology, University of Electronic Science and Technology of China Hospital, 611731, China; ^3^Department of Dermatovenereology, West China Hospital of Sichuan University, Chengdu 610041, China

## Abstract

**Background:**

Thrombin could elicit degranulation of mast cells involved in numerous physiologic and pathologic processes; however, the detailed scrutiny of this procedure and further research of possible cell signaling pathways are lacking.

**Methods:**

P815 mouse mast cells were exposed to various concentrations of thrombin for 16 h. Expression of protease-activated receptor (PAR)1, PAR2, PAR3, and PAR4 mRNA in P815 was analyzed by quantitative real-time PCR (qRT-PCR) and the fittest concentration of thrombin was decided. Then, secretions of mediators from P815 stimulated by thrombin 0.2 U/ml were determined using enzyme-linked immunosorbent assay (ELISA) and Luminex liquichip; the possible cell signaling pathways were measured by immunoblotting. Furthermore, inhibition of thrombin inhibitor (hirudin), PAR1 inhibitor (SCH79797), and MAPK inhibitors (SB203580, PD98059, and SP600125) on the mediator section was evaluated by ELISA and Luminex liquichip.

**Results:**

Thrombin 0.2 U/ml induced the elevated expression of PAR1, PAR2, PAR3, and PAR4, as well as the increasing level of phospho-I*κ*B*α*, phospho-SAPK/JNK MAPK, phospho-P38 MAPK (Thr180/Tyr182), and phospho-ERK1/2 MAPK (p44/42) in P815. Secretion of vascular endothelial growth factor (VEGF), tumor necrosis factor-*α* (TNF-*α*), interleukin- (IL-) 2, IL-6, chemokine ligand- (CCL-) 2, chemokine (C-X-C motif) ligand- (CXCL-) 1, and CXCL-5 from P815 increased apparently; this effect could be diminished by hirudin, whereas SCH79797 and MAPK inhibitors (SB203580, PD98059, and SP600125) diminish the secretions with weaker effect.

**Conclusion:**

We found the expression of PAR mRNA in P815, activation of signaling pathways of nuclear factor-kappaB (NF-*κ*B), and mitogen-activated protein kinases (MAPKs) including C-Jun NH2-terminal kinase (JNK), P38, and extracellular signal-regulated kinase 1/2 (ERK1/2), and the release of multiple inflammatory mediators stimulated by thrombin, as well as the inhibition of the inflammatory releases by hirudin, SCH79797, and MAPK inhibitors including SB203580, PD98059, and SP600125.

## 1. Introduction

Degranulation and secretion of different vasoactive and proinflammatory mediators from mast cells were accepted to be associated with multiple allergic and inflammatory diseases [1]. This process can be elicited by a number of factors including antigen (allergen), anaphylatoxins, and even physical stimuli (as reviewed in [1, 2]). Due to its crucial role in physiological and pathological functions, mast cells were broadly investigated. It is more interesting that some diseases like inflammatory bowel disease (IBD) [3] and chronic spontaneous urticaria (CSU) [4], which presented the major feature of mast cell activation, are concomitant with significant thrombin generation. Recent evidence confirmed that thrombin was not just a clotting proteinase but seemed to play crucial roles involving inflammation, allergy, and many pathophysiologic functions [5, 6]. The relationship between mast cell activity and thrombin generation may be crucially intertwined. Razin and Marx [7] discovered that thrombin could trigger mast cell degranulation rapidly without 5-lipoxygenase system activation. Pervin et al. [8] found that thrombin could induce degranulation of the mast cell depending on the calcium, but the possible cell signal pathway was not explored further. It has been reported that thrombin induced IL-6 but not TNF-*α* secretion from mouse mast cells by the thrombin receptor and Fc*ϵ*RI signaling pathway [9]. Vliagoftis reported that thrombin-elicited mast cells adhered to fibronectin via the protease-activated receptor 1 (PAR1) [10]. Evidence was accumulated that thrombin could induce directly the section of mediators from mast cells. However, the detailed scrutiny of mediators released from mast cells with the challenge of thrombin is lacking, as well as the possible cell signal pathway involved in this procedure.

PARs were a new subfamily of G protein-coupled receptors (GPCRs) and employed a crucial effect in inflammation and immune response [11]. Thrombin manipulated the conjunct inflammation-coagulation effect possibly by activation of PAR1 [6, 12]. The nuclear factor-*κ*B (NF-*κ*B) family plays a key role in responding to inflammation and immunity when inhibitors of NF-*κ*B (I*κ*B*α*), which remain as inactive NF-*κ*B proteins in the cytoplasm, become phosphorylated [13]. It has been reported that I*κ*B*α* might participate in the degranulation of mast cells by activating the NF-*κ*B way [14]. Mitogen-activated protein kinase (MAPK) families perform important effects on cell physiology [15] and have been confirmed to be associated with mast cell degranulation [16]. Based on those research of potential thrombin receptors and the signaling pathway, we hypothesise that they might be involved in the process of thrombin-activated mast cells.

In the present study, we evaluated the detailed secretion of mediators from mouse mast cell line P815 after treatment with thrombin. Then, we studied the possible receptors and cell signaling pathways associated with this procedure. The effect of various inhibitors was also presented for further research.

## 2. Materials and Methods

### 2.1. Reagents and Cells

P815 cell lines were obtained from the Shanghai Cell Bank of Chinese Academy of Sciences (Shanghai, China). SCH79797, PD98059, SB203580, and SP600125 and human antibodies against *α*-tubulin, *β*-actin, total I*κ*B*α*, total ERK1/2, total P38, and total JNK were purchased from Sigma-Aldrich (St. Louis, MO, USA). CCK8 and IL-13 ELISA kits were obtained from Boster Biological Technology (Wuhan, China). LTB4 and PGD2 ELISA kits were from Cayman (USA). The PAF ELISA kit was from the Jiancheng Bioengineering Institute (Nanjing, China). Luminex liquichip kits for VEGF, TNF-*α*, IFN-*γ*, IL-2, IL-4, IL-6, IL-10, IL-12, IL-17, CCL-2, CCL-5, CXCL-1, CXCL-2, and CXCL-5 were from Millipore (USA). Antibodies against phospho-I*κ*B*α*, phospho-SAPK/JNK MAPK, phospho-P38 MAPK (Thr180/Tyr182), and phospho-ERK1/2 MAPK (p44/42) were from GeneBioscience (Chengdu, China). Other reagents like salt or buffer components were analytical grade and were from Sigma-Aldrich (St. Louis, MO, USA). Thrombin was purchased from Sigma-Aldrich (T5772, race: rat, and 10602400001, race: human, specific activity: 20 units/31.25 mg) and dissolved in PBS with different concentrations.

### 2.2. Cell Culture

P815 cells were cultured in 75 cm^2^ tissue culture flasks in a humidified environment containing 5%CO_2_/95% air at 37°C. Cells were maintained in ATCC complete medium including IMDM (SH30228.01, HyClone) supplemented with 4 mM L-glutamine, 4.5 mg/ml glucose, 1.5 mg/ml sodium bicarbonate, 10% fetal bovine serum (FBS), 100 U/ml penicillin, and 100 *μ*g/ml streptomycin. P815 cells were maintained in a serum-free basal medium for 6 h and washed twice. At 1 × 10^6^ cells/ml concentration, P815 cells were exposed to the following challenges: various concentrations of thrombin (0 U/ml, 0.2 U/ml, 2 U/ml, 10 U/ml, and 20 U/ml), thrombin 0.2 U/ml+hirudin 10 *μ*g/ml, thrombin 0.2 U/ml+SCH79797 1.0 *μ*M, thrombin 0.2 U/ml+SB203580 10 *μ*M, thrombin 0.2 U/ml+SP600125 10 *μ*M, and thrombin0.2 U/ml+PD98059 10 *μ*M.

### 2.3. Cell Assays

**Figure 1:**
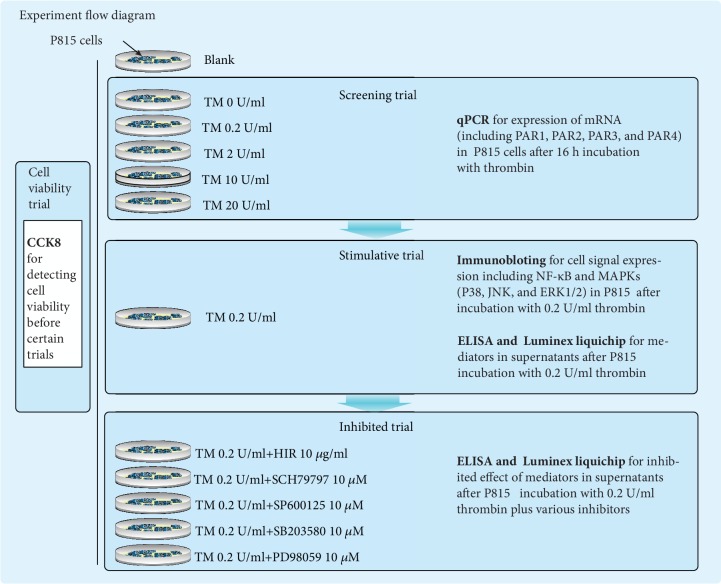
Cell experiment flow diagram. TM: thrombin; HIR: hirudin; SCH79797: PAR1 inhibitor; SP600125: JNK inhibitor; SB203580: P38 MEPK inhibitor; PD98059: ERK1/2 MAPK inhibitor; qPCR: quantitative real-time PCR; ELISA: enzyme-linked immunosorbent assay. Screening trial for deciding the fittest concentration of thrombin. Stimulative trial: thrombin 0.2 U/ml was used to stimulate P815 cells. Inhibited trial: thrombin 0.2 U/ml plus different inhibitors were used to stimulate P815 cells. Blank: P815 cells were cultured in normal condition with no challenge. We chose thrombin 0.2 U/ml as the fittest concentration by screening trial. All challenges underwent pretrial by CCK8 to exclude the interference of P815 cell death.

Cell experiments include the cell viability trial and certain cell experiments. The scenario is presented in Figure 1.

#### 2.3.1. Cell Viability Was Detected by CCK8 Kit

For detecting cell viability, P815 cells were transferred in 96 wells in triplicate at a density of 10,000/ml and cultured with a different challenge for 24 h at 37°C. Ten *μ*l CCK8 was added into each well and incubated for 90 min. Spectrophotometry at 450 nm was used to measure the OD. Relative cell viability (%) = (OD_treated_ − OD_blank_)/(OD_control_ − OD_blank_) × 100%.

#### 2.3.2. Using Quantitative Real-Time PCR (qRT-PCR) to Detect the Expression of PAR1, PAR2, PAR3, and PAR4 in P815 Cell after Challenges

After 16 h incubation of P815 cells with diverse concentrations of thrombin, the culture plates were centrifuged with 450 g for 10 min at 4°C. Cells were harvested for qRT-PCR. Total RNA was isolated by TRIzol agent; the cDNA was synthesized by the reverse transcription kit (Fermentas, Vilnius, Lithuania). The gene expression analysis was performed using Maxima™ SYBR Green qPCR Master Mix (Fermentas, Vilnius, Lithuania) as per the manufacturer's introduction. Relative mRNA expressions were standardized to the internal control GAPDH by the 2^-*ΔΔ*Ct^ cycle threshold method. The qRT-PCR primers were PAR1 (Ms) (forward: 5′-CTT CAC TTG CGT GGT CATT TGG-3′, reverse: 5′-TGG CAG GTG GTG ATG TTG AGT C-3′), PAR2 (Ms) (forward: 5′-TCC TTA CTG CAT CTG CCT ACG TG-3′, reverse: 5′-CCA GCA CGG TGA TGA TGA GTC-3′), PAR3 (Ms) (forward: 5′-CAA CTG GGT ATT TGG CGA GGT C-3′, reverse: 5′-GCT TCT GGT ATG TGA AAG GGT GAG-3′), PAR4 (Ms) (forward: 5′-TCC TCA GAC AAG CCT AAT CCA CG-3′, reverse: 5′-CAC AGC CAC CAC AAG CCC AT-3′), and GAPDH (Ms) (forward: 5′-CGG AGT CAA CGG ATT TGG TC-3′, reverse: 5′-CGG TGC CAT GGA ATT TGC CA-3′) (mouse (Ms)).

#### 2.3.3. Enzyme-Linked Immunosorbent Assay (ELISA) for Detecting the Mediator Level of LTB4, PGD2, PAF, and IL-13 in Supernatants

After 16 h incubation of P815 cells with 0.2 U/ml thrombin plus various treatments, the culture plates were centrifuged with 450 g for 10 min at 4°C. The concentrations of LTB4, PGD2, PAF, and IL-13 in the supernatants were measured by means of ELISA, according to the manufacturer's instructions. Concentrations are shown as pg/mg of protein.

#### 2.3.4. Using Luminex Liquichip to Measure the Level of VEGF, TNF-*α*, IL-17, IFN-*γ*, IL-2, IL-12, IL-4, IL-6, IL-10, IL-13, CCL-2, CCL-5, CXCL-1, CXCL-2, and CXCL-5 in Supernatants

After 16 h incubation of P815 cells with 0.2 U/ml thrombin plus different challenges, the culture plates were centrifuged with 450 g for 10 min at 4°C. For detection of the level including VEGF, TNF-*α*, IL-17, FN-*γ*, IL-2, IL-12, IL-4, IL-6, IL-10, IL-13, CCL-2, CCL-5, CXCL-1, CXCL-2, and CXCL-5 in supernatants, Luminex liquichip assays were used in accordance with the manufacturer's instructions.

#### 2.3.5. Immunoblotting for Cell Signaling Expression including I*κ*B*α* and MAPKs (JNK, P38, and ERK1/2)

After P815 cells were stimulated with thrombin 0.2 U/ml for 0.5 h, 1 h, 2 h, and 4 h, cells were washed twice using ice-cold PBS, then were systematically supplemented in a 200 *μ*l mixture of RIPA buffer (Cell Signaling Technology, #9806, 1 : 10), protease inhibitor cocktail, and phosphatase inhibitor cocktail (Cell Signaling Technology, 5872s, 1 : 100), following sonicating in a 4°C water bath (10 s, 2 times). Each sample was centrifuged at 13,000 g for 10 min to move cell debris. Total protein concentration was evaluated by a BCA kit (Beyotime, p0010) according to the manufacturer's instruction. Lording buffer (Beyotime, p0015, 1 : 5) and RIPA buffer were complemented to standardize the protein concentration in each sample. Mixtures were heated to 100°C for 10 min. An equal volume (100 *μ*g) of protein was fractionated by SDS-PAGE on a 10% acrylamide gel and transferred onto a polyvinylidene difluoride (PVDF) membrane with a Bio-Rad semidry transfer system, according to the manufacturer's instructions. After blocking with 5% milk in TBST for 1 h, membranes were immunoblotted with a human antibody against *α*-tubulin (1 : 2000 dilution), *β*-actin (1 : 2000 dilution), phosphorylated-I*κ*B*α* (1 : 1000 dilution), phosphorylated-SAPK/JNK MAPK (1 : 1000 dilution), phosphorylated-P38 MAPK (Thr180/Tyr182) (1 : 1000 dilution), phosphorylated-ERK1/2 MAPK (p44/42) (1 : 5000 dilution), total JNK (1 : 1000 dilution), P38 (1 : 3000 dilution), and ERK1/2 (1 : 2000 dilution) overnight followed by incubation with horseradish peroxidase- (HRP-) conjugated secondary antibodies. Immunoreactive bands were visualized using enhanced chemiluminescence reagents (wbkls0500, Millipore) according to the manufacturer's protocol. Densitometry analysis of immunoblots was carried out using NIH Image lab (Bio-Rad). The relative levels of protein were expressed as the ratio to *α*-tubulin or *β*-actin.

### 2.4. Statistical Analysis

All data was presented as mean ($\bar{x}$) and standard deviation (SD) if the data was normally distributed; Student's *t*-test or one-way ANOVA was used to test the differences between or among the groups. Data analysis was performed using SPSS 20.0 software (Chicago, IL, USA). *P* < 0.05 was considered statistically significant.

## 3. Results

### 3.1. Cell Viability Was Similar in P815 Cell with Various Challenges

Cell count was performed and then calculated in percentages compared to the blank group (Table 1). There are nonsignificant differences in cell viability among each group (*P* > 0.05). Those results that hunt the difference of outcomes in the following experiments were not due to the death of P815 cells with various challenges.

### 3.2. Expression of PAR1, PAR2, PAR3, and PAR4 in P815 Cells Incubated with Different Concentrations of Thrombin

**Figure 2:**
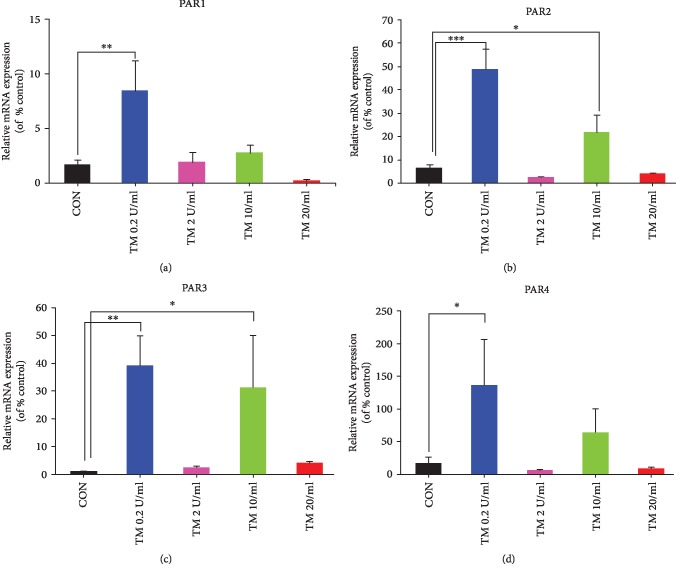
Expression of PAR1, PAR2, PAR3, and PAR4 in P815 cells after 16 h stimulation with the diverse concentration of thrombin. P815 cells were stimulated by various concentrations of thrombin at 37°C for 16 h. The expression of PAR1 (a), PAR2 (b), PAR3 (c), and PAR4 (d) mRNA in P815 cells was determined by qRT-PCR. Each trial was repeated three times. ANOVA was performed. Multiple comparisons were applied to compare the difference among the four groups. ∗ indicates that the difference between the control group and the diverse concentration of thrombin was statistically significant (^∗^*P* < 0.05; ^∗∗^*P* < 0.01; ^∗∗∗^*P* < 0.001). CON: control groups. P815 cells were incubated with an equal volume vehicle. GAPDH expression was the folding control.

Compared with the control groups, the expressions of PAR1, PAR2, PAR3, and PAR4 in groups incubated with 0.2 U/ml thrombin were all apparently elevated. The expression of PAR2 and PAR3 was increased in the group with 10 U/ml thrombin (*P* < 0.05), but there was no statistically significant difference in the group with 2 U/ml thrombin and 20 U/ml (Figure 2).

Those outcomes indicated that 0.2 U/ml thrombin may be made for treatment in certain experiences. We choose 0.2 U/ml thrombin as the fittest challenge concentration in further trials.

### 3.3. Effect of 0.2 U/ml Thrombin on Mediators' Secretion from P815 Cells

**Figure 3:**
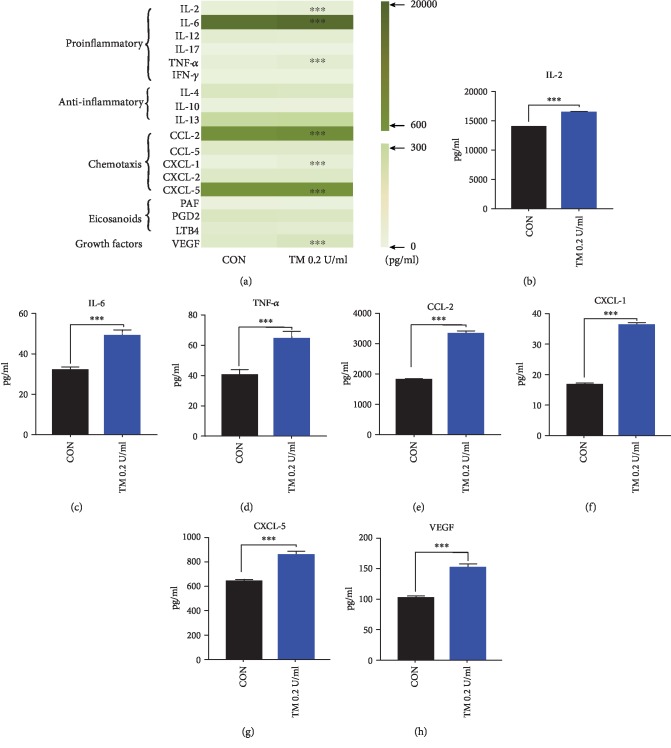
Effect of 0.2 U/ml thrombin on mediators' secretion from P815 cells. Compared with the control group, it is significantly increased in the level of IL-2 (b), IL-6 (c), TNF-*α* (d), CCL-2 (e), CXCL-1 (f), CXCL-5 (g), and VEGF (h) in supernatants after 16 h incubation with 0.2 U/ml thrombin. (a) Results of stimulation by 0.2 U/ml thrombin; data which are shown by the average outcome came from three independent trials. CON: control group; TM: thrombin. Each experiment was performed three times. Unpaired *t*-test was used to test the statistic difference between the control and trial groups; *P* < 0.05 was considered statistically different.

It was found that 0.2 U/ml thrombin could induce significant increase in secretion of VEGF, TNF-*α*, IL-2, IL-6, CCL-2, CXCL-1, and CXCL-5 from P815 cells after 16 h incubation (Figure 3), but not in PAF, LTB4, PGD2, IL-17, IFN-*γ*, IL-12, IL-4, IL-10, IL-13, CCL-5, and CXCL-2.

### 3.4. Phosphorylation of I*κ*B*α* and MAPKs (including JNK, P38, and ERK1/2) in P815 Cells Induced by 0.2 U/ml Thrombin

**Figure 4:**
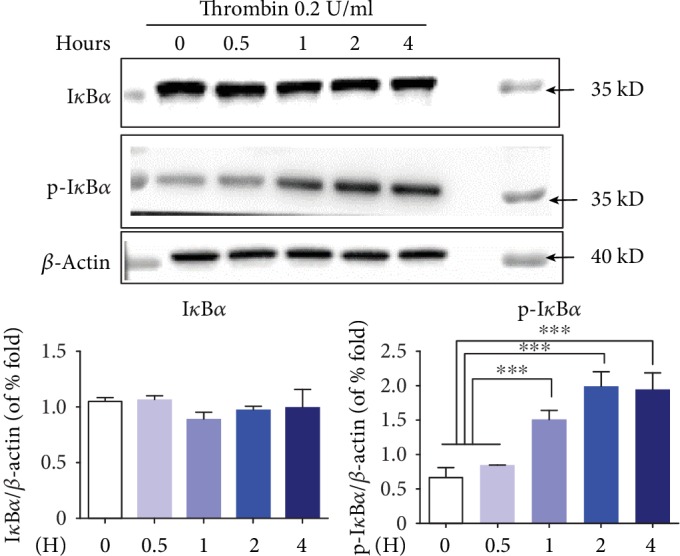
Expression of I*κ*B*α* and phospho-I*κ*B*α* in P815 cells at 0.5, 1, 2, and 4 h after treatment with 0.2 U/ml thrombin. The expression of phospho-I*κ*B*α* in P815 cells increased obviously at 1, 2, and 4 h after incubation with 0.2 U/ml thrombin but not at the level of total I*κ*B*α*. ANOVA was performed. Multiple comparisons were applied to compare the difference between each time. ∗ indicates that the difference between the control group and the diverse concentration of thrombin was statistically significant (^∗^*P* < 0.05; ^∗∗^*P* < 0.01; ^∗∗∗^*P* < 0.001).

NF-*κ*B has been recognized as a crucial component in lots of immune and inflammatory processes; the nuclear activation of NF-*κ*B depended largely on the phosphorylation of I*κ*B*α* [4]. At 1 h, 2 h, and 4 h after 0.2 U/ml thrombin treatment, expression of phosphorylated I*κ*B*α* in P815 cells was observed as obviously increased but not that of total I*κ*B*α* (Figure 4).

**Figure 5:**
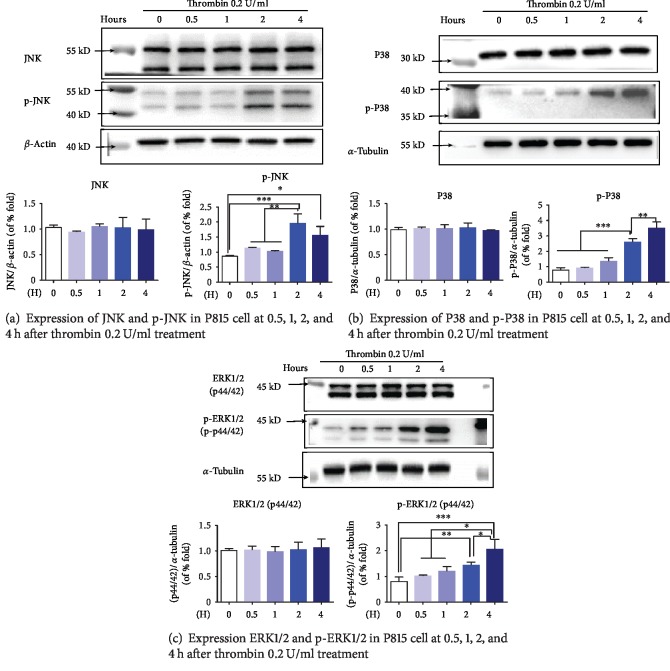
Expression of MERKs and phospho-MAPKs (including JNK, P38, and ERK1/2) in P815 cells at 0.5, 1, 2, and 4 h after stimulation of 0.2 U/ml thrombin. The expression of p-P38, p-JNK, and p-ERK1/2 MERK in P815 cells increased obviously at 2 and 4 h after incubation with 0.2 U/ml thrombin, but not in the level of total P38, JNK, and ERK1/2. Blots are standardized to *α*-tubulin or *β*-actin expression. ANOVA was performed. Multiple comparisons were applied to compare the difference between each group. ∗ indicates that the difference between the control group and the diverse concentration of thrombin was statistically significant (^∗^*P* < 0.05; ^∗∗^*P* < 0.01; ^∗∗∗^*P* < 0.001). (a) Expression of JNK and phospho-JNK, standardized by *β*-actin. (b) Expression of P38 and phospho-P38, standardized by *α*-tubulin. (c) Expression of ERK1/2 and phospho-ERK1/2, standardized by *α*-tubulin.

MAPK activity had been reviewed as a crucial role in inflammation and immunity [17]. To assess whether thrombin could lead to MAPK activation, P815 cells were examined at 0.5 h, 1 h 2 h, and 4 h after thrombin 0.2 U/ml stimulation. Thrombin 0.2 U/ml enhanced phosphorylation of JNK, P38, and ERK1/2 at 2 h and 4 h after treatment, whereas no apparent change in the level of total MAPKs was found at any time point after incubation (Figure 5).

### 3.5. Effect of the Thrombin Inhibitor, PAR1 Inhibitor, and Cell Signaling Inhibitors on Thrombin-Induced Releasing of Mediators of P815 Cell

We employed hirudin (thrombin inhibitor), SCH79797 (PAR1 inhibitor), SP600125 (a JNK pathway inhibitor), SB203580 (P38 MAPK selective inhibitor), and PD98059 (ERK1/2 MAPK inhibitor) to further evaluate the possible thrombin signaling pathways in P815 cells.

**Figure 6:**
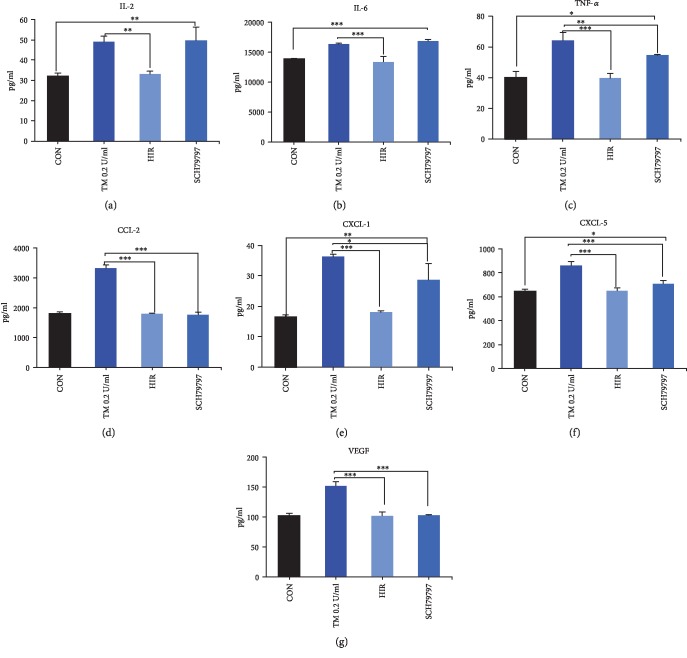
Effect of thrombin inhibitor and PAR1 inhibitor on thrombin-induced releasing of mediators of P815 cell. Thrombin inhibitor (hirudin) diminished secretion of mediators from P815 cells. Compared with TM 0.2 U/ml group, PAR1 inhibitor (SCH79797) decreased secretions of TNF-*α* (c), CCL-2 (d), CXCL-5 (e), CXCL-1 (f), and VEGF (g), but not of IL-2 (a) and IL-6 (b). Compared with the control group, expression of IL-2 (a), IL-6 (b), TNF-*α* (c), CXCL-1 (e), and CXCL-5 (f) remained increased in the SCH79797 groups. ANOVA was performed. Multiple comparisons were applied to compare the difference between inhibited groups and the thrombin group or control group. ∗ means that the difference was statistically significant between groups; ^∗^*P* < 0.05, ^∗∗^*P* < 0.01, and ^∗∗∗^*P* < 0.001. Difference between the control group and the thrombin group is shown in Figure 3. Difference between the control group and the hirudin group was not statistically different. CON: control group; TM: thrombin.

Hirudin diminished thrombin-induced secretion of mediators from P815 cells. Compared with the TM 0.2 U/ml group, the PAR1 inhibitor (SCH79797) decreased secretions of VEGF, TNF-*α*, CCL-2, CXCL-5, and CXCL-1, but not of IL-2 and IL-6 (Figure 6). Compared with the control groups, secretions of TNF-*α*, IL-2, IL-6, CXCL-5, and CXCL-1 remained increasing in the SCH79797 groups. Those results indicated that secretion of mediators of P815 induced by thrombin depended partially on PAR1.

**Figure 7:**
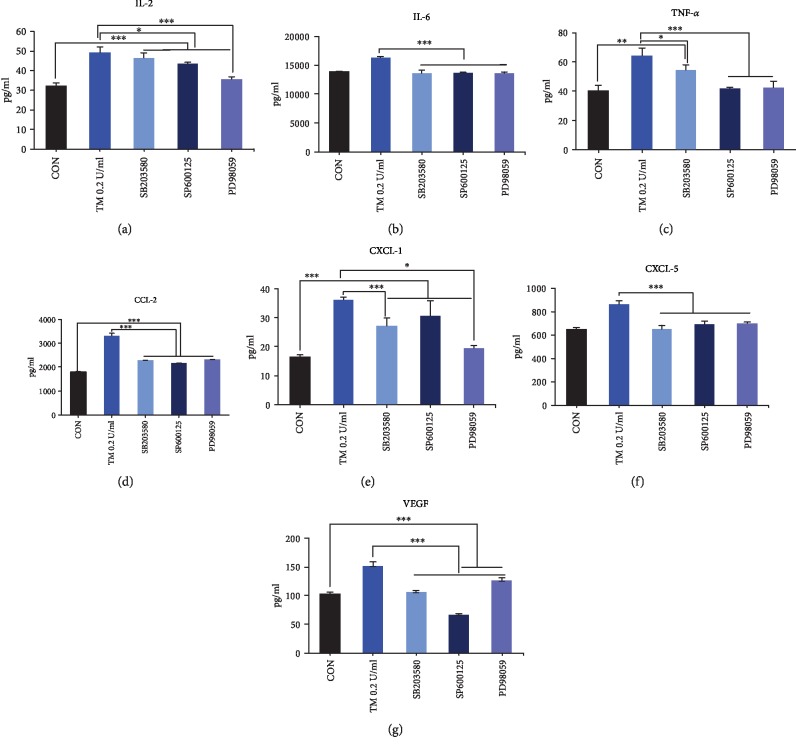
Effect of cell signaling inhibitors including selective P38 MAPK inhibitor (SB203580), ERK1/2 MAPK inhibitor (PD98059), and JNK pathway inhibitor (SP600125) on thrombin-induced releasing of mediators of P815 cells. Selective P38 MAPK inhibitor (SB203580), ERK1/2 MAPK inhibitor (PD98059), and JNK pathway inhibitor (SP600125) lessen, but not completely, the mediators released by P815 cells induced by thrombin. ANOVA was performed. Multiple comparisons were applied to compare the difference between cell signaling inhibited groups and the thrombin group or control group. ∗ means that the difference was statistically significant between groups; ^∗^*P* < 0.05, ^∗∗^*P* < 0.01, and ^∗∗∗^*P* < 0.001. Difference between the control group and the thrombin group is shown in Figure 3. CON: control group; TM: thrombin.

SB203580, SP600125, and PD98059 lessen mediators released from P815 cells induced by thrombin (Figure 7), which appear to show that the process of thrombin stimulating P815 cells occurs partly through activation of JNK, P38, and ERK1/2 MAPK signaling pathways.

## 4. Discussion

In our present study, we found the expression of PAR mRNA in mast cell P815, activation of signaling pathways of NF-*κ*B and MAPKs including JNK, P38, and ERK1/2, and the release of multiple inflammatory mediators stimulated by thrombin, as well as the inhibition of the inflammatory releases by the thrombin inhibitor (hirudin), PAR1 inhibitor (SCH79797), and MAPK inhibitors including JNK inhibitor (SP600125), P38 inhibitor (SB203580), and ERK1/2 inhibitor (PD98059).

Activated PARs combine with diverse G proteins and trigger signal transduction pathways resulting in the rapid modulation of the secretion of mediators involved in inflammation. Four members of PAR1, PAR2, PAR3, and PAR4 have been confirmed so far. Activation of PARs might be associated with mast cell degranulation [18]. It has been reported that PAR1, PAR3, and PAR4 could be activated by thrombin [19, 20]. In Chinese hamster ovary cells, PAR2 seems to resist thrombin [21]. However, in this study, PAR1, PAR2, PAR3, and PAR4 in P815 cells seemed to all be activated by 0.2 U/ml thrombin. Because of the important role of PAR2 in immunity and inflammation [11], we determined the concentration of 0.2 U/ml thrombin for further research, although thrombin 0.2 U/ml may only represent one section of the process with thrombin stimulating the mast cell. In our supplemental material (supplement [Supplementary-material supplementary-material-1] and [Supplementary-material supplementary-material-1]), we make a superficial exploration and find that the time frame and the concentration frame of this process are more complicated.

Mast cells were often supposed as the first-line reaction cells for immunological invaders due to their ubiquity in the surface in contact with the external environment [22]. Mast cells could be activated by lots of subjects including stress and inflammation [23], then they secrete various mediators with or without degranulation. In spite of the well-known activated method of mast cells was the autoantibody-induced trigger which represented the major pathogenic mechanism of many allergic diseases and inflammation; nonallergic activated triggers for mast cells were also involved in many diseases [1]. Under different activation, mediators released from mast cells diverge significantly. Of importance, mast cells could produce both proinflammatory and anti-inflammatory effects after activation [22]. These cytokines released from mast cells were functional and potential therapeutic [24–26]. Vergnolle et al. demonstrated that thrombin function had not only proinflammatory but also anti-inflammatory effects [27]. Our results revealed that 0.2 U/ml thrombin could elicit obviously increased secretion of proinflammation mediators including IL-2, IL-6, and TNF-*α* from P815 cells but not in anti-inflammatories such as IL-4, IL-10, and IL-13. This difference might due to our dose of thrombin being lower with 0.2 U/ml. Our results indicated that a low dose of 0.2 U/ml thrombin may only exert a proinflammatory effect.

The activation of mast cells caused the release of chemokine and arachidonic mediators, which both possess potent inflammatory activity [28]. Chemokine released from activated mast cells might potentially exert both an autocrine and a paracrine effect to adjacent cells under inflammation [29]. Arachidonic acid can induce, amplify, or dampen inflammatory responses and regulate immune responses [30]. VEGF is the powerful mediator of angiogenesis, and mast cells releasing VEGF were linked with allergic inflammation like asthma [31]. We explored with interest whether those mediators were produced from mast cells during the process of thrombin stimulation. Our finding of obvious increase of VEGF and chemokines including CCL-2, CXCL-1, and CXCL-5 but not arachidonic mediators indicated the importance of preventing chemokines and growth factors in clinical practices. Although it has been proved that thrombin was able to induce the activation of mast cells and IL-6 secretion [7–9], our research provided the detailed investigation of the process.

Type 1 immune response is defined as the increased levels of IFN-*γ* and/or IL-12 while a type 2 immune response is the character of an elevated level of IL-4, IL-6, IL-10, and/or IL-13. In addition to helper T (Th) lymphocytes, mast cells were the sources of these cytokines [32]. Dysregulation of type 1 and type 2 cytokines is involved in many human infectious and inflammatory diseases. We detected the representative type 1 cytokine IFN-*γ* and type 2 cytokine IL-4. The result implicated that mast cells might not be involved in immune response during the process of 0.2 U/ml thrombin-induced activation. In the clinical practice, 0.2 U/ml thrombin may be the initial stage of “allergic-like” diseases which is relevant with the activation of mast cells with detectable evidence of thrombin-induced, but with the absence of allergen-specific, IgE antibodies. Our research might perform more efficient and precise therapeutic strategies for those diseases.

It was well known that NF-*κ*B and MAPK cell signaling pathways play an important role in the process of inflammation and immunity [17, 33]. NF-*κ*B and MAPK cell signaling pathways may be the downstream pathway of activated PARs [11]. We checked those pathways to confirm our hypothesis. Our results indicated that NF-*κ*B and MAPK cell signaling pathways were involved in the secretion of mediators induced by thrombin. Those activated pathways may be cross-linked not only by thrombin but also by others like histamine [34]. Considering the outcomes in inhibited trials, in which inhibition of the inflammatory releases by the MAPK inhibitors including JNK inhibitor (SP600125), P38 inhibitor (SB203580), and ERK1/2 inhibitor (PD98059) was the weaker effect, it appeared to confirm that activation of JNK, P38, and ERK1/2 MAPK signaling pathways responded partially for the section of mediators released by thrombin. Those active cell signal pathways, which were directly or indirectly stimulated by thrombin and others, might participate in the enormous complex physiological and pathological process.

In our inhibited trials, thrombin inhibitor (HIR) could significantly diminish the secretion of mediators induced by thrombin. PAR1 inhibitor (SCH79797) can inhibit the sections of mediators but not fully. Those outcomes indicated that sections induced by thrombin partially depended on activation of PAR1. PAR1 has been well accepted to regulate vascular function in the physiological and pathological process. Thrombin stimulating mast cells through PAR1 might produce pro- and anti-inflammation [27]. We also make a tentative trial to explore the effect of PAR agonists involving the process with thrombin stimulating P815; the outcomes appear to find that PAR1, PAR2, and PAR4 are involved in this process (supplementary Figures [Supplementary-material supplementary-material-1], [Supplementary-material supplementary-material-1], and [Supplementary-material supplementary-material-1]). Our observation was consistent with previous reports that PAR1 activation responded partially for the interaction between thrombin and mast cells. However, the possibility of other receptors being involved in this action could not be ruled out, particularly the obvious rise of the expression of PAR2, PAR3, and PAR4 after incubation with 0.2 U/ml thrombin. Further studies are required to fully understand the possible receptors participating in the process.

There were some methodological considerations in the present experiment. First, thrombin 0.2 U/ml may not be the best concentration for activating mast cells, which may only represent the initial stage for this complicated process. Second, the time frame and the concentration frame of the process with thrombin stimulating mast cells may be very complex. We explore it superficially. In our supplement (supplement Figures [Supplementary-material supplementary-material-1] and [Supplementary-material supplementary-material-1]), we present the outcomes of different time courses and different concentrations stimulating mast cells, which appear to reconfirm that this process is more complicated than anticipated. Cell analysis at 8 h and supernatant analysis at 16 h may only explain one section of this process. In spite of the current study being an initial trial for thrombin being involved in the degranulation of mast cells, we presented the outcomes of thrombin simulating P815 cells and researched the possible signaling pathways in this procedure, as well as the inhibited effect of various inhibitors. Our study appears to provide the optimized therapy strategy for many diseases associated with thrombin-induced mast cell activation. The coalition of antithrombin drugs was efficient for those diseases, and the anti-inflammation therapy in the initial stage was necessary. To investigate further, the next trials should be performed in human mast cell lines and animal experiments.

## 5. Conclusion

1. The expressions of PAR1, PAR2, PAR3, and PAR4 mRNA in P815 cells were increased by the stimulation of thrombin
2. The growth factor VEGF; multiple proinflammatory mediators including TNF-*α*, IL-2, and IL-6; and chemokines including CCL-2, CXCL-1, and CXCL-5 were released by the stimulation of thrombin
3. Section of inflammatory mediators could be blocked by the thrombin inhibitor hirudin and partially inhibited by the PAR1 inhibitor SCH79797
4. Phosphorylation of inhibitory NF-*κ*B and activation of P38, JNK, and ERK1/2 in the P815 cells could be induced by thrombin, and the release of inflammatory mediators could be blocked by JNK inhibitor SP600125, P38 inhibitor SB203580, and ERK1/2 inhibitor PD98059

## Figures and Tables

**Table 1:** Cell viability was evaluated by CCK8 kit.

Groups	Cell viability (%)
Blank	100.0 ± 2.8
Thrombin (0 U/ml)/control	103.6 ± 10.7
Thrombin (0.2 U/ml)	98.2 ± 10.6
Thrombin (2 U/ml)	94.8 ± 7.8
Thrombin (10 U/ml)	92.7 ± 9.1
Thrombin (20 U/ml)	91.8 ± 11.7
Thrombin (0.2 U/ml)+HIR (10 *μ*g/ml)	93.2 ± 7.7
Thrombin (0.2 U/ml)+SCH79797 (1.0 *μ*M)	92.6 ± 8.7
Thrombin (0.2 U/ml)+SP600125 (10 *μ*M)	92.5 ± 8.8
Thrombin (0.2 U/ml)+SB203580 (10 *μ*M)	91.6 ± 9.4
Thrombin (0.2 U/ml)+PD98059 (10 *μ*M)	91.9 ± 7.2

Cell count was performed in 3 separate wells of each group. ANOVA was performed to test the difference. The difference was not statistically significant among the groups (*P* > 0.05). Blank group: P815 cells were cultured in normal condition with no challenge. Control group: P815 cells were incubated with the vehicle. HIR: hirudin; SCH79797: PAR1 inhibitor; SP600125: JNK inhibitor; SB203580: P38 MEPK inhibitor; PD98059: ERK1/2 MAPK inhibitor.

## Data Availability

The data used to support the findings of this study are available from the corresponding authors upon request.

## Abbreviation

CCK8: Cell counting kit-8
CCL: Chemokine ligand
CSU: Chronic spontaneous urticaria
CXCL: Chemokine (C-X-C motif) ligand
ERK1/2: Extracellular signal-regulated kinase 1/2
GPCRs: G protein-coupled receptors
HIR: Hirudin
IL: Interleukin
I*κ*B*α*: Inhibitor kappa B*α*
JNK: C-Jun NH2-terminal kinase
LTB4: Leukotriene B4IFN-*γ* (interferon-*γ*)
MAPKs: Mitogen-activated protein kinases
NF-*κ*B: Nuclear factor-kappaB
PAR: Protease-activated receptor
TM: Thrombin
TNF-*α*: Tumor necrosis factor-*α*
VEGF: Vascular endothelial growth factor.

## Supplementary Materials

Supplementary Materials In the supplementary materials, we find that the time frame and concentration frame of the process of thrombin stimulating the P815 cell is more complicated; we explain that the use of thrombin 0.2 U/ml may be feasible but imperfect. We also present the results in agitated trials and provide evidence for our finding that TM stimulating mediator secretion of mast cells may be partly through PARs. Figure 1: the expression of mediators in supernatant at 0.5, 2, 4, 8, 12, and 16 h after P815 cells were incubated with PBS, 0.2 U/ml thrombin, 1.2 U/ml thrombin, and 2 U/ml thrombin. Figure 2: the expression of PAR in P815 at 0.5, 2, 4, 8, 12, and 16 h after P815 cells were incubated with PBS, 0.2 U/ml thrombin, 1.2 U/ml thrombin, and 2 U/ml thrombin. Figure 3: expression of CXCL-1 in supernatant at 16 h after P815 cells were incubated with PBS, TM 0.2 U/ml, A2387, TM 0.2 U/ml+TFLLR-amide, TM 0.2 U/ml+2-furoyl-LIGRLO-amide, and TM 0.2 U/ml+AYPGKF-amide. Figure 4: expression of IL-6,TNF-*α*, CCL-2, and VEGF in P815 cells at 8 h after P815 cells were incubated with PBS, TM 0.2 U/ml, A2387, TM 0.2 U/ml+TFLLR-amide, TM 0.2 U/ml+2-furoyl-LIGRLO-amide, and TM 0.2 U/ml+AYPGKF-amide. Figure 5: expression of PARs in P815 cells at 8 h after P815 cells were incubated with PBS, TM 0.2 U/ml, A2387, TM 0.2 U/ml+TFLLR-amide, TM 0.2 U/ml+2-furoyl-LIGRLO-amide, and TM 0.2 U/ml+AYPGKF-amide.
