# Supplementary material for: Thrombin Induces Secretion of Multiple Cytokines and Expression of Protease-Activated Receptors in Mouse Mast Cell Line
Source: Mediators Inflamm. 2019 Nov 14;2019:4952131. doi: 10.1155/2019/4952131 (PMC6878808; doi:10.1155/2019/4952131)
Supplement: Supplementary Materials — In the supplementary materials, we find that the time frame and concentration frame of the process of thrombin stimulating the P815 cell is more complicated; we explain that the use of thrombin 0.2 U/ml may be feasible but imperfect. We also present the results in agitated trials and provide evidence for our finding that TM stimulating mediator secretion of mast cells may be partly through PARs. Figure 1: the expression of mediators in supernatant at 0.5, 2, 4, 8, 12, and 16 h after P815 cells were incubated with PBS, 0.2 U/ml thrombin, 1.2 U/ml thrombin, and 2 U/ml thrombin. Figure 2: the expression of PAR in P815 at 0.5, 2, 4, 8, 12, and 16 h after P815 cells were incubated with PBS, 0.2 U/ml thrombin, 1.2 U/ml thrombin, and 2 U/ml thrombin. Figure 3: expression of CXCL-1 in supernatant at 16 h after P815 cells were incubated with PBS, TM 0.2 U/ml, A2387, TM 0.2 U/ml+TFLLR-amide, TM 0.2 U/ml+2-furoyl-LIGRLO-amide, and TM 0.2 U/ml+AYPGKF-amide. Figure 4: expression of IL-6,TNF-α, CCL-2, and VEGF in P815 cells at 8 h after P815 cells were incubated with PBS, TM 0.2 U/ml, A2387, TM 0.2 U/ml+TFLLR-amide, TM 0.2 U/ml+2-furoyl-LIGRLO-amide, and TM 0.2 U/ml+AYPGKF-amide. Figure 5: expression of PARs in P815 cells at 8 h after P815 cells were incubated with PBS, TM 0.2 U/ml, A2387, TM 0.2 U/ml+TFLLR-amide, TM 0.2 U/ml+2-furoyl-LIGRLO-amide, and TM 0.2 U/ml+AYPGKF-amide. [file 4952131.f1.docx]

**Supplemental material**


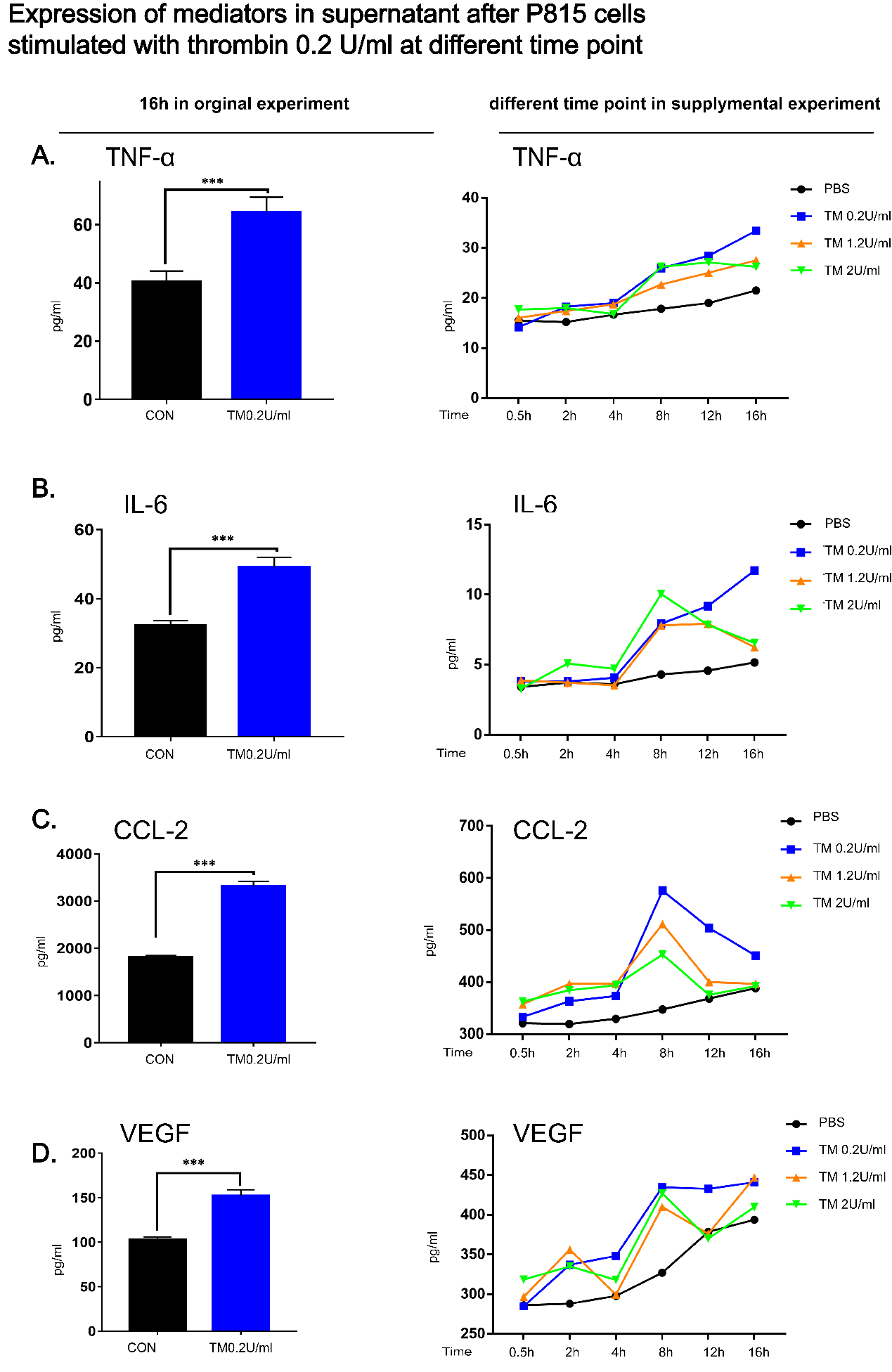


**Firgue.1. The expression of mediators in supernatant at 0.5,2,4,8,12,16h after P815 cells incubating with PBS, 0.2U/ml thrombin, 1.2U/ml thrombin and 2U/ml thrombin**

Left: data came from orginal experiment. P815 cell incubated with 0.2 U/ml thrombin. Supernatant was collect after 16h incubating. Detailed information was listed in revised manuscript Figure.3.

Right:data came from supplemental experiment. P815 cell incubated with 0.2 U/ml,1.2U/ml,2u/ml thrombin and PBS. Supernatant was collected at 0.5h,2h,4h,8h and 16h after incubating. Data in duplicate came from one experiment. Average digit was extacted and presented on left figure. The outcomes appear to recomfirm the process of thrombin stimulating mast cell is more complex and interesting.

TM: thrombin. Of note, thrombin in supplemental experiment came from Roche (10602400001)


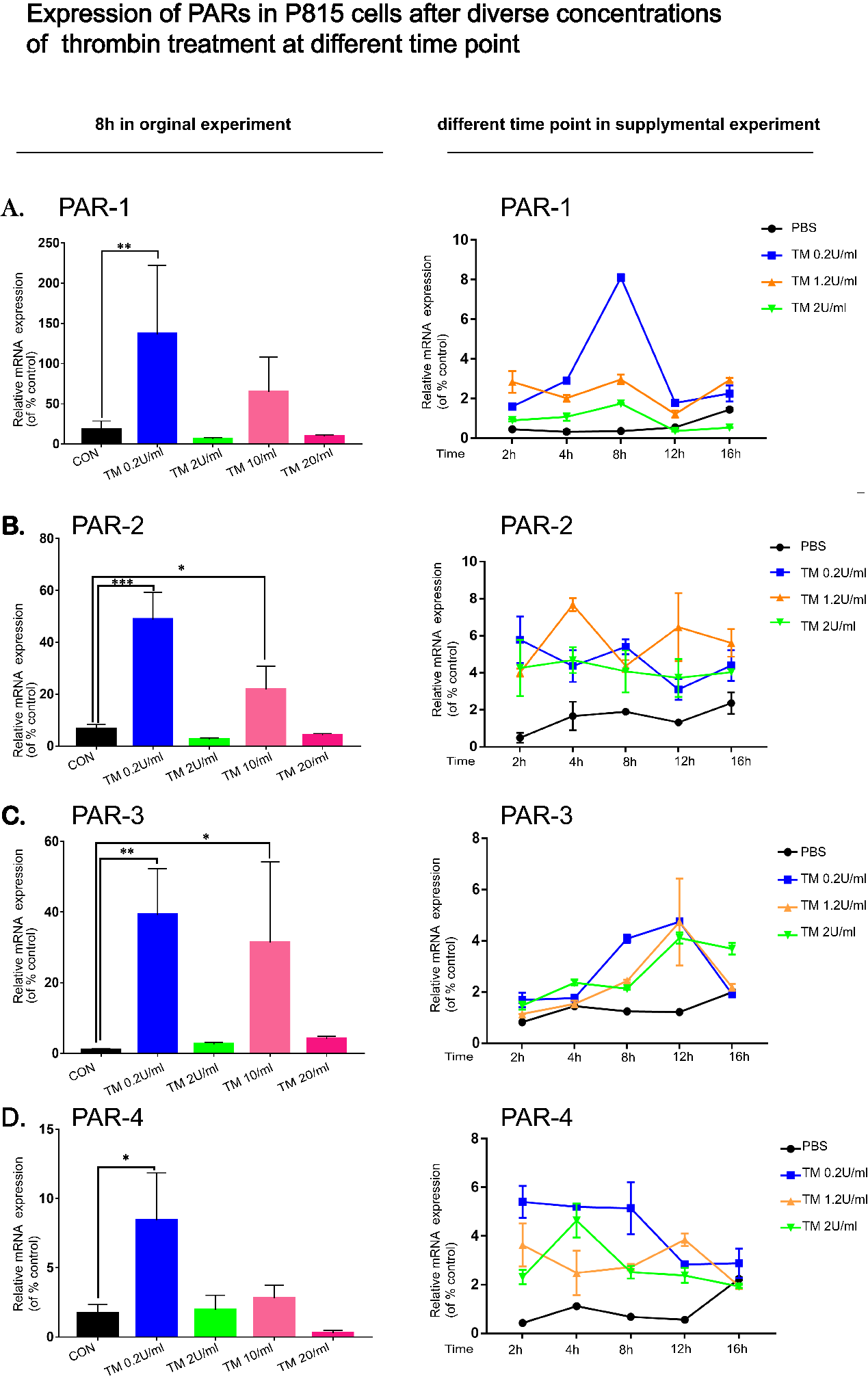


**Firgue.2. The expression of PAR in P815 at 0.5,2,4,8,12,16h after P815 cells incubating with PBS, 0.2U/ml thrombin, 1.2U/ml thrombin and 2U/ml thrombin**

Left: data came from orginal experiment. P815 cell incubated with 0.2 U/ml thrombin. Cells were collect after 8h incubating. Detailed information was listed in revised manuscript Figure.2.

Right:data came from supplemental experiment. P815 cell incubated with 0.2 U/ml,1.2U/ml,2u/ml thrombin and PBS. Supernatant was collected at 0.5h,2h,4h,8h and 16h after incubating. Data in duplicate came from one experiment. Average digit was extacted and presented on left figure. Expression of PARs did not increase with increasing concentration of thrombin, nor with extension of incubated time. The outcomes appear to recomfirm the process of thrombin stimulating mast cell is more complicated.

TM: thrombin. Of note, thrombin in supplemental experiment came from Roche (10602400001)

**
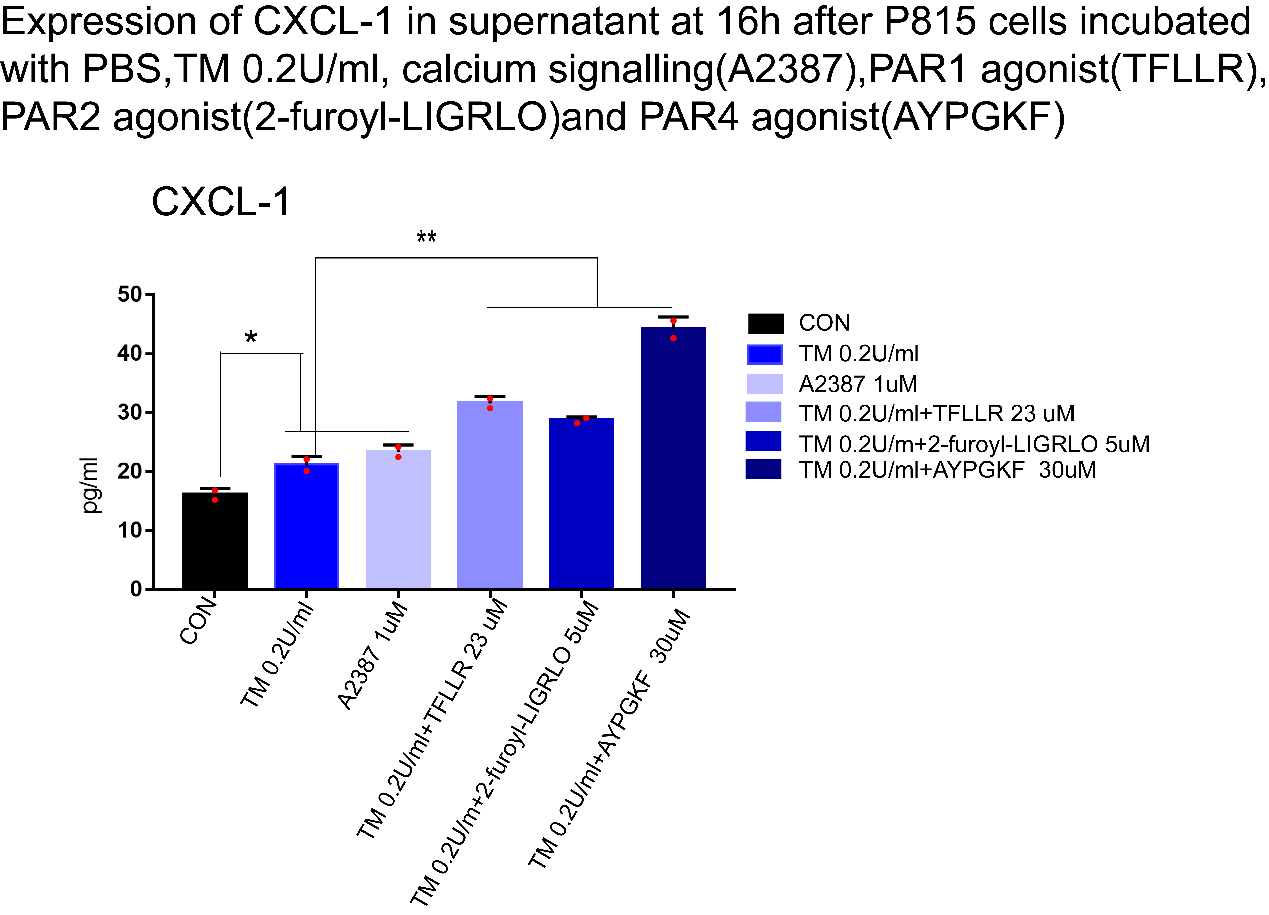
**

**Figure.3. Expression of CXCL-1 in supernatant at 16h after P815 incubating with PBS, TM** **0.2U/ml, A2387, TM 0.2U/ml +TFLLR-amide; TM 0.2U/ml +2-furoyl-LIGRLO-amide and TM 0.2U/ml +AYPGKF-amide**

Data came from supplemental experiment. P815 cell incubated with PBS, TM 0.2U/ml +calcium signalling(A2387 1uM), TM 0.2U/ml +PAR1 agonist(TFLLR-amide 23uM), TM 0.2U/ml + PAR2 agonist(2-furoyl-LIGRLO-amide 5uM)and TM 0.2U/ml + PAR4 agonist(AYPGKF-amide 30uM). Supernatant was collected at 16h after incubating. CXCL-1 Elisa kit was used to detect the expression of CXCL-1 in supernatant. Data in duplicate came from one experiment. Red dots represent the detailed digits. Ordinary one-way analysis was performed. Multiple comparisons were applied to compare the difference among groups of PBS,TM and A2387(or PARs agonist). * indicated the difference among groups was statistically significant (*, P<0.05; **, P<0.01).CON: control groups, P815 cells were incubated with equal volume vehicle. Compared with CON groups, the expression of CXCL-1 increases in TM and A2387 groups. Compared with TM groups, the expression of CXCL-1 increases in TM plus TFLLR-amide, TM plus 2-furoyl-LIGRLO-amide and TM plus AYPGKF-amide groups. TM: thrombin. Of note, thrombin in supplemental experiment came from Roche (10602400001)

**
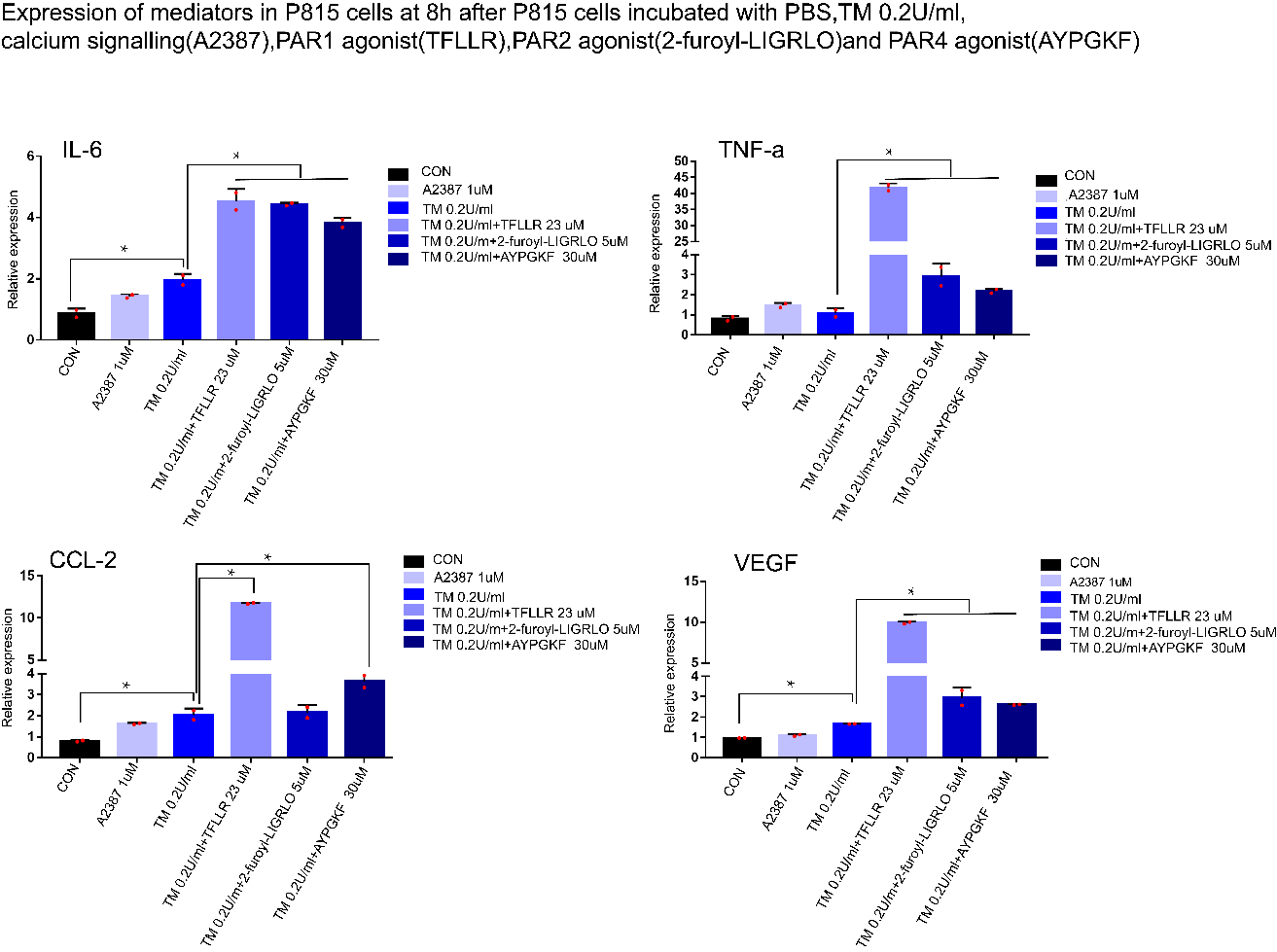
**

**Figure.4. Expression of IL-6,TNF-ɑ,CCL-2 and VEGF in P815 cells at 8h after P815 incubating with PBS, TM 0.2U/ml, A2387, TM 0.2U/ml +TFLLR-amide; TM 0.2U/ml +2-furoyl-LIGRLO-amide and TM 0.2U/ml +AYPGKF-amide**

Data came from supplemental experiment. P815 cell incubated with PBS, TM 0.2U/ml +calcium signalling(A2387 1uM), TM 0.2U/ml +PAR1 agonist(TFLLR-amide 23uM), TM 0.2U/ml + PAR2 agonist(2-furoyl-LIGRLO-amide 5uM)and TM 0.2U/ml + PAR4 agonist(AYPGKF-amide 30uM). Cells were collected at 8h after incubating. qPCR was used to measure the expression of IL-6,TNF-ɑ,CCL-2 and VEGF in P815 cells. Data in duplicate came from one experiment. Red dots represent the detailed digits. Ordinary one-way analysis was performed. Multiple comparisons were applied to compare the difference among groups of PBS,TM and each PARs agonist. * indicated the difference among groups was statistically significant (*, P<0.05).CON: control groups, P815 cells were incubated with equal volume vehicle. Compared with TM groups, the expression of IL-6,TNF-ɑ, and VEGF increases in PARs agonist groups. Expression of CCL-2 increases in TM plus TFLLR-amide and TM plus AYPGKF-amide groups.

**
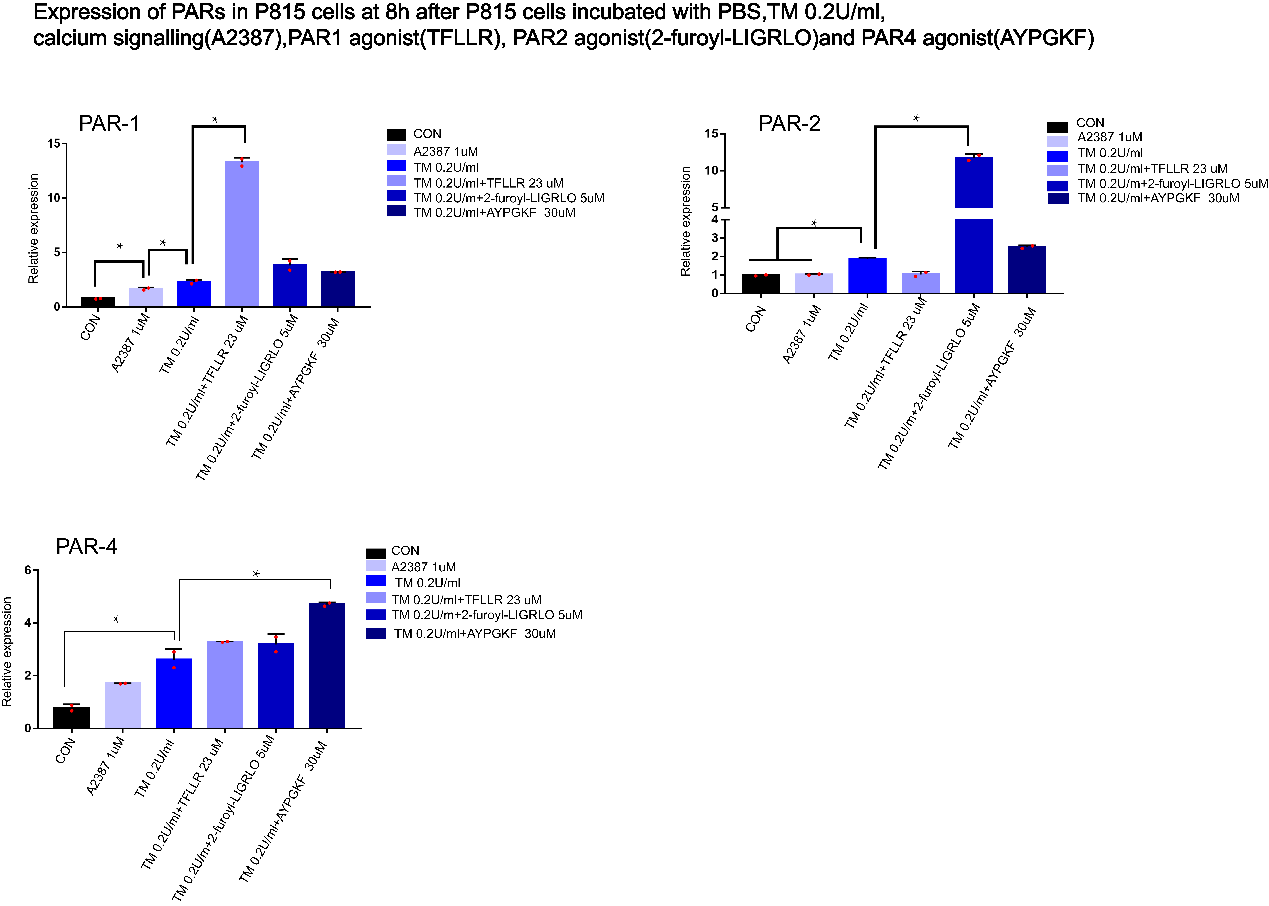
**

**Figure.5. Expression of PARs in P815 cells at 8h after P815 incubating with PBS, TM 0.2U/ml, A2387, TM 0.2U/ml +TFLLR-amide; TM 0.2U/ml +2-furoyl-LIGRLO-amide and TM 0.2U/ml +AYPGKF-amide**

Data came from supplemental experiment. P815 cell incubated with PBS, TM 0.2U/ml +calcium signalling(A2387 1uM), TM 0.2U/ml +PAR1 agonist(TFLLR-amide 23uM), TM 0.2U/ml + PAR2 agonist(2-furoyl-LIGRLO-amide 5uM)and TM 0.2U/ml + PAR4 agonist(AYPGKF-amide 30uM). Cells were collected at 8h after incubating. qPCR was used to measure the expression of PAR1,2 and 4 in P815 cells. Data in duplicate came from one experiment. Red dots represent the detailed digits. Ordinary one-way analysis was performed. Multiple comparisons were applied to compare the difference among groups of PBS,TM and each PARs agonist. * indicated the difference among groups was statistically significant (*, P<0.05).CON: control groups, P815 cells were incubated with equal volume vehicle. Compared with TM groups, the expression of PAR1 increases in group with TM 0.2U/ml +PAR1 agonist(TFLLR-amide 23uM). The expression of PAR2 increases in TM 0.2U/ml + PAR2 agonist(2-furoyl-LIGRLO-amide 5uM) groups. The expression of PAR2 increases in TM 0.2U/ml + PAR4 agonist(AYPGKF-amide 30uM).
